# Aphasia therapy on a neuroscience basis

**DOI:** 10.1080/02687030701612213

**Published:** 2008-05-21

**Authors:** Friedemann Pulvermüller, Marcelo L. Berthier

**Affiliations:** Medical Research Council Cognition and Brain Sciences Unit, Cambridge, UK; Centro de Investigaciones Médico-Sanitarias (CIMES), University of Malaga, Spain

**Keywords:** Aphasia, Chronic aphasia, Constraint-induced therapy, Intensive language action therapy (ILAT), Language, Laterality, Motor system, Neuro imaging, Neurotransmitters, Perisylvian cortex, Pharmacotherapy

## Abstract

**Background:**

Brain research has documented that the cortical mechanisms for language and action are tightly interwoven and, concurrently, new approaches to language therapy in neurological patients are being developed that implement language training in the context of relevant linguistic and non-linguistic actions, therefore taking advantage of the mutual connections of language and action systems in the brain. A further well-known neuroscience principle is that learning at the neuronal level is driven by correlation; consequently, new approaches to language therapy emphasise massed practice in a short time, thus maximising therapy quantity and frequency and, therefore, correlation at the behavioural and neuronal levels. Learned non-use of unsuccessful actions plays a major role in the chronification of neurological deficits, and behavioural approaches to therapy have therefore employed shaping and other learning techniques to counteract such non-use.

**Aims:**

Advances in theoretical and experimental neuroscience have important implications for clinical practice. We exemplify this in the domain of aphasia rehabilitation.

**Main Contribution:**

Whereas classical wisdom had been that aphasia cannot be significantly improved at a chronic stage, we here review evidence that one type of intensive language-action therapy (ILAT)—constraint-induced aphasia therapy—led to significant improvement of language performance in patients with chronic aphasia. We discuss perspectives for further improving speech-language therapy, including drug treatment that may be particularly fruitful when applied in conjunction with behavioural treatment. In a final section we highlight intensive and rapid therapy studies in chronic aphasia as a unique tool for exploring the cortical reorganisation of language.

**Conclusions:**

We conclude that intensive language action therapy is an efficient tool for improving language functions even at chronic stages of aphasia. Therapy studies using this technique can open new perspectives for research into the plasticity of human language circuits.

Around one third of stroke patients develop a language disorder and the incidence of aphasic language disturbances is estimated to lie at around 3000 per million inhabitants (0.37%), which is twice that of Parkinson’s disease (Elman, Ogar, & Elman, 2000; Simmons-Mackie, Code, Armstrong, Stiegler, & Elman, 2002). This amounts to 150,000–300,000 patients with aphasia in countries such as Spain, UK, France, Ukraine, Congo, or Argentina. Because of their communication disability, the majority of patients with chronic aphasia are unable to maintain their previous job and suffer from a reduction of their social contacts. It is therefore evident that the disorder aphasia causes great problems at individual, social and socio-economic levels.

As research in the cognitive neuroscience of language has recently made great progress and led to substantial advances in our understanding of language processes in the human brain, one may rightly ask what consequences such knowledge increase might have for improving life conditions of patients with aphasia. This article will therefore highlight some findings from neuroscience and discuss their implications for language therapy and clinical practice. We will also ask whether the success of such new therapy approaches has been well documented. In this context it will become apparent that even patients with chronic aphasia, having suffered from this disease for many years, can achieve improvement of their condition by participating in language training on a neuroscience basis. Thus we conclude that application of neuroscience knowledge in aphasia therapy can be beneficial at present, and we also point to new perspectives where insights from neuroscience might become therapeutically fruitful in the future.

The reverse issue—whether the investigation of language therapy and the related restitution of brain processes can help answer critical questions in modern neuroscience research—will also be addressed: Chronic patients participating in intensive treatment can be investigated using neuroimaging technology before and after a short treatment interval. Changes in these chronic patients’ language-related brain activation over treatment can provide unique clues about cortical reorganisation processes related to language, which are not available from studies of patients during stages of spontaneous recovery. Clearly, new insights from language plasticity at chronic stages critically depend on the availability of a therapy regime that is effective at chronic stages.

## Neuroscience Knowledge and its Implications For Aphasia Therapy

For more than 150 years neurologists, psychologists, and linguists have been investigating language disorders caused by stroke and other diseases of the brain. Over this period not only has our wisdom about human language and communication greatly improved, but the available knowledge about the brain and its functioning has also increased dramatically. There is now a substantial body of knowledge about how neurons function and communicate with each other. We also know specific features of the connectivity of cortical areas and nerve cells therein. It has also become clear which mechanisms in the brain make it possible for us to learn and to change our behaviours. The effect of drugs on both neural function and neural learning has been investigated meticulously. Perhaps most importantly, we now know from modern neuroimaging studies which cortical areas become active when language is being processed and we even know features of the time course with which neuronal populations in different areas ignite during language production and comprehension. We will highlight here a few important pieces of neuroscience knowledge that, we believe, are of relevance for designing and planning language therapy in the clinic.

### Coincidence and correlation learning

Neurophysiological research demonstrates a relationship between functional changes in neurons and the learning of new information and behaviours by the individual. If two things happen at the same time in the environment, the individual may learn that the two belong together. The brain basis for this might be what is sometimes called Hebbian learning: nerve cells that fire together also wire together. When two neurons are frequently active at the same time, the connection between them becomes stronger (Hebb, 1949). This is called coincidence learning. Cells that are active independently of each other do not become associated, or even weaken their links (Artola & Singer, 1993; Tsumoto, 1992). This synaptic weakening implies that, in addition to coincidence mapping, the correlation of neuronal activity determines connection strengths. Correlation and coincidence mapping can even be sensitive to the timing of activation in the millisecond range, so that precise temporal sequences of neuronal firing are mapped on directed connections between nerve cells (Bi, 2002; Froemke & Dan, 2002; Gutig, Aharonov, Rotter, & Sompolinsky, 2003). Such synaptic modification provides an important neural basis of learning: As two co-occurring objects are being connected in the individual’s mind, neurons that respond to features of these objects become bound together functionally in the individual’s brain.

What are the implications of correlation and coincidence learning for the processes of language learning, or relearning and restitution, in the brain of an aphasic patient? Due to the brain lesion, some of the neurons important for processing language, words, their relationship to each other, and their meanings, have been deleted, disconnected, or otherwise functionally impaired (Dell, Schwartz, Martin, Saffran, & Gagnon, 1997; Harley, 1996; Plaut, 1996; Pulvermüller & Preissl, 1991). Also the connections between word representations, and between word and meaning representations, may have become so weak that it is no longer possible to find the right word for an object or action, or to continue a sentence in an appropriate manner. Setting up new links and strengthening remaining ones in the lesioned neuronal populations might therefore be important for regaining functionality. Correlation and coincidence learning can contribute to such brain repair at the functional level and, ultimately, strengthening of links driven by correlation should benefit behavioural performance.

How can a high correlation between relevant neuronal activations be achieved in aphasia therapy? Obviously, the more frequently two relevant brain events occur together, the more the critical connections will be strengthened. Assuming that aphasia therapy can induce the relevant coincidence of neuronal firing, this implies that more training will help more. Also the correlation between two activation events in the brain becomes stronger the more frequently the two events occur together. However, the correlation is diminished, and may go down to zero, if the two events appear independently of each other. In a therapy environment, it is possible to create optimal learning conditions by using given words primarily in the context of well-defined objects, actions, and other words. However, if the patient returns home, or to his or her normal environment, and interacts with others, such high correlation between words, objects, and actions might not be preserved. At the neural level, this implies that if, in the therapy session, some neuronal strengthening takes place, contrarian weakening will result from the interaction outside therapy. Such weakening can be avoided if therapy is conducted in a rather intensive manner, with many therapy hours following each other as closely as is possible and feasible. In this case, the time span during which a strong correlation is present is being maximised, whereas the intervening time without such correlation is minimised. If, after a successful therapy period, functional connections between word, object, and action representations are successfully re-established in the patient’s brain, it is no longer necessary to maintain the strong correlation in the environment, as word, object, and action representations can call each other up through their strong mutual links.

From this discussion it emerges that, given that correlation and coincidence learning represent a neuroscience principle, it is advisable to administer language therapy in a massed-practice fashion. It has recently been recommended to administer as much as 3 hours of language therapy per day for weeks in patients with chronic aphasia who are in a stable condition (Pulvermüller et al., 2001). In theory, if the patient’s condition allows for it, even higher therapy frequencies might be beneficial. Massive practice of several hours per day for prolonged periods was mentioned in the early days of aphasia therapy (e.g., Luria, 1970; Wepman, 1953), but its influence had not been examined systematically then. High therapy quantity and frequency, administering a maximum of training in as short a period as possible, might lead to better treatment outcome, not only relative to less therapy but, critically, also compared with the same amount of therapy given in a sparse manner, for example, with one therapy hour a week spread out over several weeks or even months. As the prediction is that the more one practices in a short time, the better the outcome will be, we propose the following therapy principle:

*Massed practice principle:* It is advantageous to maximise quantity (number of therapy hours) and frequency (number of therapy hours per time) of language therapy.

It is important to note that the high frequency and quantity principle has two implications. The first is that, generally, more training has a benefit over less training, or no training at all. This may appear as a near-trivial statement today, but it is noteworthy that not long ago some scholars argued that aphasia therapy was entirely ineffective (Lincoln et al., 1984). Ironically, this conclusion had been based on therapy given with very low frequency. Meanwhile there is unambiguous evidence for the effectiveness of aphasia therapy and for the claim that a larger amount of therapy results in greater benefits (Basso, 2005; Basso, Capitani, & Vignolo, 1979; Bhogal, Teasell, & Speechley, 2003; Hinckley & Carr, 2005; Hinckley & Craig, 1998; Holland, Fromm, DeRuyter, & Stein, 1996; Raymer, Kohen, & Saffell, 2006; Robey, 1994, 1998). This quantity (“more helps more”) effect must be distinguished from the effect of frequency, implying that, given the number of therapy hours is kept constant, a large amount of therapy in a short time interval is more efficient than the same amount spread out over longer periods of time. This latter frequency effect of equal quantities of treatment has been substantiated by recent work (Pulvermüller et al., 2001). However, it should also be mentioned here that, although it is scientifically proven that aphasia therapy is to the benefit of the patient, especially if applied in high quantity and with high frequency, the practical implementation, in many cases, falls short of the desiderata and requirements (Katz et al., 2000).

### Language–action links

In one view, language resides in encapsulated modular processing systems, each specialised for one type of linguistic information. Accordingly, a processor for speech sounds would be largely independent from a module for syntax or sentence processing, and processors for lexical information and semantic meaning would likewise each be housed in separate informationally encapsulated systems (e.g., Friederici, 2002; Harley, 2001; Levelt, Roelofs, & Meyer, 1999; Shelton & Caramazza, 1999). All these processors would further be placed in a super-system for language, which, after stimulation, would process linguistic information autonomously for a significant period of time, without information exchange with perceptual systems or with those brain parts involved in action execution.

Modular theories of language had important consequences for aphasia therapy. As the idea had been that language subsystems were separate, the prevalent idea was to practise syntax, semantics, phonology, and other linguistic information processing separately in linguistic tasks. Rules of syntax and phonotactics, single words and semantic distinctions were all practised separately, without embedding their application into interaction schemes where words and sentences have their normal function in interaction and communication (for discussion, see Pulvermüller, 1990; Pulvermüller & Roth, 1991). The proposal had been that only after prolonged periods of structural exercises should additional training aim at transferring linguistic skills to conversation contexts.

Recent neuroscience evidence has made some modular views on language difficult to maintain. Neuroimaging studies have demonstrated that, when words and sentences are being recognised and understood, not only the classical language centres in left perisylvian cortex are activated, but also a range of additional brain areas that are normally involved in action and perception processes. When hearing speech or reading words, the motor system is automatically facilitated so that motor responses are easier to elicit (Fadiga, Craighero, Buccino, & Rizzolatti, 2002; Watkins, Strafella, & Paus, 2003). Magnetoencephalography demonstrated that perception- and action-related brain parts become active near simultaneously in language comprehension (Pulvermüller, Shtyrov, & Ilmoniemi, 2003). When language is being comprehended the motor system activation even reveals information about action-related meaning of the language material processes (Pulvermüller, 2005; Shtyrov, Hauk, & Pulvermüller, 2004; Tettamanti et al., 2005). Spoken language sounds, when heard, activate motor areas specifically involved in processing motor movements required to produce these same sounds (Pulvermüller et al., 2006).

**Figure 1.**
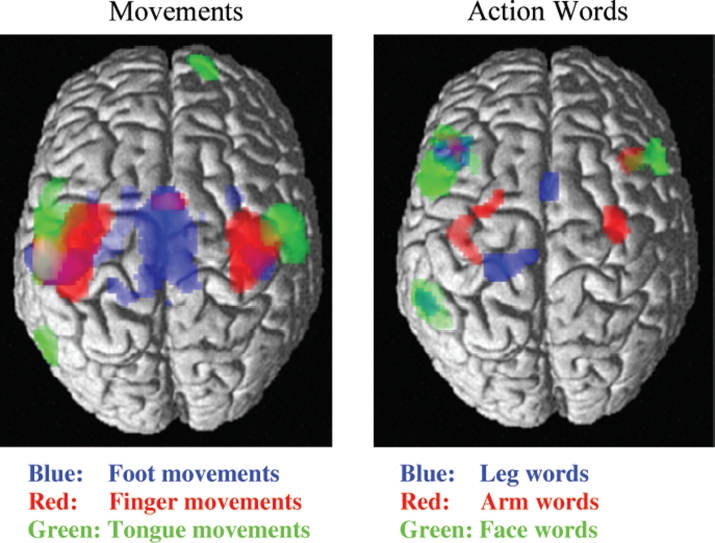
Neurofunctional links between language and action: Left: Somatotopic sensorimotor activation in pre- and postcentral gyrus during simple repetitive movements of the tongue (in green), index finger (red), and foot (blue). Right: Somatotopic activation during passive reading of action words related to the face (e.g., “lick”, in green), arm or hand (“pick”, red), and leg or foot (“kick”, blue). The somatotopic activation of motor systems reflects aspects of the meaning of the language elements under processing (from Hauk et al., 2004).

For example, in one study it was found that the referential meaning of action verbs was reflected by motor activation (Hauk, Johnsrude, & Pulvermüller, 2004). Words related to different parts of the body such as *lick, pick*, and *kick* activated those brain parts that usually control the actions indicated by the words’ meaning. Leg motor areas were activated by words such as “kick”, arm and hand areas were activated by words like “pick”, and action words semantically related to the face and articulators such as “lick” activated brain areas around the locus controlling tongue movements (Figure 1, diagram on the right). This motor activation in language processing happens extremely rapidly, within 100–200ms after a spoken word can be understood from the input (Pulvermüller, Shtyrov, & Ilmoniemi, 2005c; Shtyrov et al., 2004). This demonstrates a link, at the level of the cortex, between language and action processors (Pulvermüller, 2005; Rizzolatti & Craighero, 2004). The old view that language and action are each situated in separate encapsulated modules, which process their information for a significant amount of time without talking to each other, can therefore not be maintained (for further discussion, see Barsalou, Kyle Simmons, Barbey, & Wilson, 2003; Glenberg & Kaschak, 2002). Corresponding neuroscience evidence exists for the rapid functional interaction and information exchange between the brain systems for language and visual perception (Moscoso Del Prado Martin, Hauk, & Pulvermüller, 2006; Pulvermüller & Hauk, 2006). In sum, there is ample evidence that perception and action systems coactivate and interact with the language system, especially in semantic processing.

The tight and rapid link between language and action processes at the level of the cortex has implications for language therapy. If language and action representations are strongly connected with each other—or, to use a particularly plastic phrase once coined by the philosopher Ludwig Wittgenstein, language “is woven into” actions (Wittgenstein, 1953)—it will be possible to activate one by activating the other. If this view is correct, the deficit caused by a lesion in the cortical areas for language production and comprehension might therefore be counteracted by activating action circuits. Such action activation might lead to facilitatory activation in language areas. Such facilitation could, in principle, be beneficial in the language production and comprehension process. Critically, the links between action and language representations seem to be extremely specific, as the action word experiments demonstrate (Pulvermüller, 2005).

In summary, if the cortical language system is tightly interwoven with the cortical action system, action context may facilitate language processes. We therefore propose as a further principle of aphasia therapy grounded in neuroscience research the following principle:

*Behavioural relevance principle:* It is advantageous to practise language in relevant action contexts.

### The aggravating role of learned non-use

Stroke and other brain diseases lead to destruction of nervous tissue and therefore to behavioural deficits, for example aphasia or motor deficits. One may therefore conclude that all features of a chronic motor impairment or language deficit are a direct consequence of the physical lesion of nerve cells. But this is not the case. Other important mechanisms at the behavioural and neuronal levels are also at work. An important mechanism is that of learned non-use. When the afferent, sensory fibres of an arm are cut so that an animal, or human, is unable to experience sensations in that arm, the individual tends to stop using that extremity (Taub, Ellman, & Berman, 1966; Taub, Heitmann, & Barro, 1977; Taub, Perrella, & Barro, 1973). This may be due to depression of central nervous system motor activity due to the reduction of sensory input. At an early stage, this reduction of central motor activity in the motor cortex makes it impossible for the individual to move the limb. However, after some time, recovery of functions has progressed so far that the individual would, in principle, be able to use the extremity once again. However, at that stage, and even though all motor fibres are fully intact, the individual does not usually regain control over the limb. Critical for this is a learning process occurring during the time of factual motor impairment. The monkey, or human, has learned that after the injury it (he/she) is less likely to succeed with motor movement of the impaired arm. Errors, and therefore punishment, are more likely to occur and therefore the individual stops making attempts using that extremity. At the same time use of the intact arm is fully accurate, and therefore successful and reinforcing for the individual. The individual therefore acquires a habit of replacing movements of the impaired extremity with movements of the intact one. In other words, the individual has learned not to use the impaired extremity. And this is precisely the reason why even after functional recovery of the motor system, the animal or human would still fail to make appropriate use of the affected arm (Taub, Uswatte, & Elbert, 2002).

A similar process is likely to take place after brain lesions such as those caused by stroke. The motor system is lesioned, for example the arm movements become impaired; thus actions performed with that arm become less accurate. This failure leads to absence of reward or direct punishment, and this reduces the likelihood of the arm being used in future. If this learned non-use becomes established and permanent there will be a functional impairment even after the tissue has partly recovered and some reorganisation processes have taken place (Taub et al., 2002). As it is known that functional reorganisation depends on practice (e.g., Hamzei, Liepert, Dettmers, Weiller, & Rijntjes, 2006; Liepert et al., 2000), it is well possible that learned non-use also hinders reorganisation processes essential to functional recovery. This applies to motor movement deficits and may also be relevant at higher cognitive levels.

In the case of language impairments caused by stroke and other brain lesions, a process of learned non-use is clearly evident as well: Patients in many cases avoid expressions with which they have systematic problems. Clearly, some features of aphasias are a direct causal consequence of neuronal dysfunction, and may restrict patients to a very limited set of utterances, a single recurring utterance in the extreme, or to phonologically distorted jargon. However, other features of aphasias may critically depend on the application of specific strategies. Patients with some remaining but imperfect language skills may actively retreat to simplistic utterances or even avoid verbal communication, replacing speaking and writing by gestures and pointing. In the worst case, the patient retreats from social interaction and avoids communication entirely, or reduces it to the absolute minimum. These undesirable facets of learned non-use of language have a clear correlate at the level of nerve cell activity.

The interplay between neurofunctionally determined behavioural deficit and strategic behavioural change resulting in learned non-use is perhaps best exemplified using a much discussed type of aphasic speech: agrammatism with telegraphic style. A causal origin of this deficit may be a specific processing impairment for certain kinds of lexical elements—especially grammatical function words and inflectional affixes (Caplan, 1982, 1987; Goodglass, 1997; Kolk, van Grunsven, & Keyser, 1985; Pulvermüller, 1995). However, as a consequence of that deficit, and after realising it and its consequences, many patients tend to change their communication strategies and avoid the use of grammatical words, thus speaking in a telegraphic style similar to that used by normal speakers when writing telegrams (Kolk & Heeschen, 1990). In this case, the neurofunctional impairment in function word processing, which implies difficulties and lack of success in function word production, leads to the application of a strategy of learned non-use to function words and certain sentence structures. The late and slow emergence of agrammatism reported recently in a case study (Code, Müller, Tree, & Ball, 2006) is consistent with the view that learning plays a major role in the emergence of this disturbance.

It is clear that the nerve cell populations that normally contribute to verbal communication become less used when the patient avoids speaking, or neglects certain parts of speech, sentence forms, or types of communications. One may argue that the major cause of the patient’s avoidance of verbal interaction is, in fact, the brain lesion. However, as argued earlier (Kolk & Heeschen, 1990), there is usually a gap between what is still possible, with some effort and some risk of failure, and what the patient is usually ready to perform. In order to reactivate and therefore possibly strengthen those language circuits that have survived a lesion, it is necessary to push the patient to his or her linguistic and communicative limits. In other words, it is important to constrain verbal communication so that the patient takes advantage even of those residual language skills he or she would normally not risk applying in everyday language interaction due to fear of failure. In language therapy, it is possible to introduce so-called constraints that force the patient to use those residual language skills, therefore pushing the individual to their verbal and communicative limits. This was emphasised in the context of an approach called constraint-induced aphasia therapy (Pulvermüller et al., 2001). As the word “constraint” can be misunderstood in the negative sense of “restraint”, it may appear more appropriate to speak about “focusing” in this context: The idea is to help the patient focus on those language tools that are in their range of capabilities although they frequently remain unused, thereby enriching the tools and forms of communication in which the patients participate (see also later discussion).

In summary, it appears that learned non-use is an important factor in behavioural deficits arising from cortical lesions. In order to overcome the undesirable consequences of learned non-use it is imperative to use constraints, or focusing tools, in language therapy that direct the patient towards using his or her remaining but unused language and verbal skills. Only then is it possible to reactivate and reorganise language circuits that would normally not be used because of risk of verbal failure in interactions. From here we deduce a third principle of aphasia therapy:

*Focusing principle:* It is advantageous to focus patients on their remaining language abilities, especially on those they avoid using

## Neuronal Models of Language and its Functional Reorganisation

Aphasia therapy may profit from brain models of language. Such models, specifying language at the level of mechanistic neuronal circuits that need to be repaired, may be a guide to designing new forms of neurorehabilitation. Clearly it is possible to approach aphasia therapy on the basis of purely abstract cognitive models that do not specify details of neuronal circuitry. However, as detailed knowledge of the engine, carburettor, and transmission may be a better starting point for repairing a car than abstract knowledge about its components, so it might be equally advantageous to use a neurofunctional model when designing and planning neurocognitive therapy. Admittedly, different models are under discussion in the neuroscience of language and by selecting one we may therefore err, at least with regard to the fine details distinguishing between them. However, as there is now substantial convergence on the basis of unambiguous evidence, for example, regarding the wide distribution of brain circuits for language and their intrinsic connections with other cognitive, perceptual, and action systems, it is plausible that aspects of current models reflect aspects of the truth. Therefore, applying these brain models of language to practical domains, especially aphasia therapy, may well be fruitful.

### Action-perception networks for spoken word forms

Speaking a word is caused by neuronal activity in the motor cortex. Motor cortex is controlled by pre-motor and inferior prefrontal areas anterior to it, where Broca’s area is localised. Near simultaneously with the motor activation, the production of a word form leads to a speech signal, which activates the auditory system and leads to neuronal activity in superior temporal cortex, in the auditory cortex, and adjacent areas in superior temporal gyrus, Wernicke’s area. As these activations in inferior frontal cortex and superior temporal cortex happen near simultaneously, the part- taking neurons may strengthen their mutual connections. There are long-distance links between inferior frontal cortex and superior temporal cortex, and the correlation learning principle (see earlier) implies that what fires together wires together. Therefore, during speaking even of unfamiliar syllables and new word forms, neuronal representations, distributed circuits with inferior frontal and superior temporal subcomponents, and strong internal connections are likely being set up (Braitenberg & Pulvermüller, 1992; Fry, 1966; Garagnani, Wennekers, & Pulvermüller, 2007; Pulvermüller, 1999). These circuits or networks can be considered a neuronal counterpart of spoken word forms. Neurophysiological evidence for the existence of such memory traces for spoken words has been reported in a number of studies (for a recent review, see Pulvermüller & Shtyrov, 2006). Specific multimodal neuron types— namely mirror neurons contributing to action execution as well as to action perception—play an important role in the neuronal assemblies processing language and action in the human brain (Berthier, Pulvermüller, Green, & Higueras, 2006; Rizzolatti & Craighero, 2004; Rizzolatti, Fogassi, & Gallese, 2001). For processing written words, an additional area in the left fusiform gyrus is activated as well as perisylvian language circuits (for discussion, see McCandliss, Cohen, & Dehaene, 2003; Price & Devlin, 2003). As these distributed networks link information about word production (in inferior frontal cortex) and information about perceptual aspects of the word form (stored in temporal cortex), it appears appropriate to speak of action-perception networks that constitute the cell assemblies for language elements. Cortical action-perception networks of a similar kind have been documented in the animal literature (Fuster, 1997; Fuster, Bodner, & Kroger, 2000). The postulated action-perception networks bind multimodal information about the articulatory and auditory features of the spoken word form and, potentially, about the visual features of the written word form and the writing gestures necessary for writing it down.

If action-perception networks constitute the cell assemblies mechanistically processing language elements, such as single words and larger utterances, a lesion in the perisylvian language cortex of the left hemisphere implies that these networks lose some of their neurons and connections and, therefore, become functionally impaired (Garagnani et al., 2007; Pulvermüller & Preissl, 1991). Functional reorganisation is necessary to regain their functionality. In principle, such functional reorganisation can take two routes: The remaining neuronal circuits can strengthen their internal links and therefore become functional again. Or the circuit may incorporate additional neurons that could compensate, to a degree, for those lost due to the lesion. Both processes are not mutually exclusive, but possibly interact in functional reorganisation in the brain.

If, after a stroke or other brain lesion, a word-related network cannot be activated and the respective word can therefore no longer be produced or understood, it may still be possible to achieve its activation in a therapeutic setting. This can be done by stimulation through different modalities, for example by showing the written form of the word and reading it to the patient for repetition at the same time, and by a range of other language tasks. Such stimulation approaches have long been established in aphasia therapy (Luria, 1970; Rosenbek, LaPointe, & Wertz, 1995; Schuell, 1974; Weigl & Bierwisch, 1970). An additional argument provided in the neuroscience context is the following: The correlation principle (see earlier) implies that activation of these networks should frequently happen in as short a time as possible, so that intervening noise can be minimised. Therapy should take place in a massed practice fashion. In this case the functionally impaired circuits might become reorganised and repaired in the most effective manner.

### Semantic links

**Figure 2.**
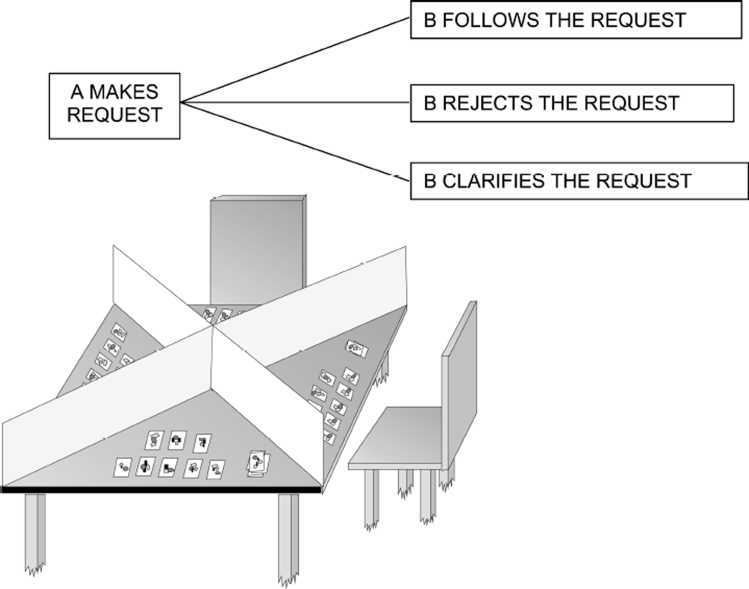
A paradigm for language action therapy: Four partners, usually three patients with aphasia and one therapist, sit around a table and have cards in front of them. Two copies of each card are in the game. There are barriers between the partners, as indicated in the bottom diagram (adapted from Neininger et al., 2004). The participants make requests to obtain a twin card for one they already have, follow requests made by others, reject requests if they cannot follow them, or ask back in case there is a comprehension problem. This interaction follows the normal sequence of these speech acts in dialogues, which is indicated schematically at the top.

When words are being used in the context of objects and actions, it is not only the language areas of the brain in perisylvian cortex that become active (see previous section). If a word systematically relates to a visually perceivable object, activation is present in perisylvian cortex because the word form is being used and, at the same time, there is activation of the visual system in inferior-temporal and occipital cortex, because the objects might be present in the environment. Even if the object is not present in a particular situation, it may be that the object representation in temporal occipital areas and the neuronal circuit representing the word in perisylvian cortex have developed links, so that one activates the other. This accounts for our experience that when hearing the word “crocodile” we cannot help thinking of the respective object. It also accounts for the findings from neuroimaging studies that word meaning relates to middle and inferior temporal activation, and that this activation can reflect specific semantic types and category specificity (Chao, Haxby, & Martin, 1999; Moscoso Del Prado Martin et al., 2006; Noppeney & Price, 2002; Pulvermüller & Hauk, 2006). In the same way, links between word form and action are being set up when, for example, the child learns that the word form “run” means a particular type of action. The sensory motor neurons in central sensorimotor cortex that play a role in controlling the running would become active near simultaneously with those neurons that store and cortically implement the word form “run”. Therefore, if we speak of running, we activate our motor system, which, so to speak, starts a virtual running action (see previous section; Boulenger, Roy, Paulignan, Deprez, Jeannerod, & Nazir, 2006; Jeannerod, 2006; Pulvermüller, 2005). And the reverse is also true. If there is activation in a specific part of the motor system, it has been found that words semantically related to the actions controlled by the brain part stimulated become activated or primed (Pulvermüller, Hauk, Nikulin, & Ilmoniemi, 2005a). Figure 2 shows a mechanistic model of specific connectivity between neuronal populations in language cortex and motor areas. These connections may underlie the cognitive links for semantic binding between action words and referential aspects of their meaning. The model receives support from a range of neuroscience experiments (see previous section).

As there is evidence for mutual connections between language and action systems, it is imperative to explore the potential usefulness of these facilitatory connections in language therapy. The model in Figure 2 implies that, if the perisylvian cortex suffers from a lesion, it is advantageous to activate the motor system, by using specific action contexts relevant to the language elements practised. In this case, specific activation can flow from the motor system to the language system in perisylvian space. The behavioural relevance principle (see earlier) commands language usage in relevant action contexts, so that the mutual links of the language and action systems can be taken advantage of. An analogous point can also be made with respect to the links between language and object perception systems (Barsalou, 1999; Pulvermüller, 1999) and for those between language and other perceptual systems (e.g., olfactory, see González et al., 2006) as well.

### The hemispheres

A remark is necessary regarding the roles of the two cerebral hemispheres in language control and recovery. It is sometimes believed that language is housed in the left or dominant hemisphere, at least in most right-handed subjects, and that the other, the right hemisphere not dominant for language, does not in any way contribute to language in these individuals. This view is not accurate, as it is well documented that the right hemisphere most significantly contributes to prosodic and emotional (Ross & Mesulam, 1979) as well as semantic-pragmatic aspects of language processing (Joanette, Goulet, & Hannequin, 1990; Kasher, Batori, Soroker, Graves, & Zaidel, 1999). Importantly, core-language functions, including lexico-semantic processing, could also be attributed to the right hemisphere. For example, research using lateralised tachistoscopic presentation (Day, 1977) and, critically, studies of split-brain patients demonstrated lexical and semantic abilities in the right, non-dominant hemisphere (Zaidel, 1976, 1983). Furthermore, behavioural studies in right-handed subjects have shown that, while the directly stimulated left hemisphere performs better than the right on perceptual language tasks (Chiarello, Nuding, & Pollock, 1988), tasks that simultaneously present words to both hemispheres lead to much improved language-processing outcomes than those directly stimulating only the dominant hemisphere (Endrass, Mohr, & Pulvermüller, 2004; Mohr, Pulvermüller, & Zaidel, 1994). This pattern of results, which could only be found for words but not for matched pseudowords, suggests that the best lexico-semantic processing system is not the left hemisphere on its own, but the coordinated interactive system composed of both hemispheres together. Corroborating evidence also comes from imaging studies and patient work. For example, the motor areas activated by action-related language were found in both hemispheres to a similar degree (Hauk et al., 2004) and lesions in the right hemisphere can lead to specific linguistic deficits only affecting certain word types (Neininger & Pulvermüller, 2003; Tranel, Damasio, & Damasio, 1997; Tranel, Logan, Frank, & Damasio, 1997). Clearly, perisylvian activation is usually lateralised to the left hemisphere, but this does not mean that only the left perisylvian cortex is active and the right hemisphere is silent, or even suppressed in its activation. It appears that the perisylvian cortex of the left hemisphere is activated strongly and there is some less pronounced activation of the right perisylvian cortex, too (Springer et al., 1999).

Taken together, a range of findings indicate that both hemispheres take their shares in language control in the intact human brain. It therefore would appear likely that both hemispheres also play a role in language recovery and reorganisation. In line with this statement, a number of studies have advocated a functional role in cortical reorganisation of language functions for the left dominant hemisphere (Heiss, Kessler, Thiel, Ghaemi, & Karbe, 1999; Price & Crinion, 2005), but other studies have argued for a significant contribution of the right dominant hemisphere (Abo et al., 2004; Blank, Bird, Turkheimer, & Wise, 2003; Musso et al., 1999; Thomas, Altenmüller, Marckmann, Kahrs, & Dichgans, 1997; Weiller et al., 1995), or have provided evidence for a reorganisational role of both hemispheres (Cardebat et al., 2003; Crosson et al., 2005; Dobel et al., 2001; Pulvermüller, Hauk, Zohsel, Neininger, & Mohr, 2005b). This pattern of results seems to be best captured by the interpretation that both hemispheres can play a role in the language recovery and reorganisation process. It may be that task-specific factors and the nature of the lesions account, in part, for why some studies found the major dynamics in only one hemisphere. For example, syntactic processes do not seem to emerge in right- hemispheric language restitution (see Dobel et al., 2001), whereas residual right- hemispheric language functions at the lexico-semantic level are clearly evident in severe forms of aphasia, for example mixed-transcortical aphasia with complete lesion of the left-perisylvian areas or hemispherectomy (Berthier, 1999; Berthier et al., 1991; Kastrau, Wolter, Huber, & Block, 2005; Pulvermüller & Schönle, 1993).

We would suggest that thinking in terms of hemispheric activation might be at too coarse a level to capture the fine details of cortical relearning. It might be more fruitful to think in terms of the activation of specific language, perception, and action circuits in aphasia therapy. Intervention strategies might sensibly target specific types of linguistic action-perception networks that are functionally impaired but can still be activated within some specific stimulation setting. Action contexts can be used to effectively stimulate the network parts in perisylvian cortex, especially in the left hemisphere and partly in the right as well. In therapeutic settings, constraints can be used to focus the patient on utterances and actions that might otherwise fall victim to learned non-use. Therapy should be massed and be applied frequently in a short time, so that the correlation of the relevant activations is high.

## Intensive Language Action Therapy: Constraint-Induced Aphasia Therapy as an Example

In the preceding sections we have highlighted neuroscientifically grounded principles for aphasia therapy and their relation to brain models of language. We have discussed the principles of massed practice, behavioural relevance, and language-action linkage, and focusing language usage on the communicative needs of the patient by application of constraints. It is a challenging enterprise to implement all of these three neuroscientifically grounded principles in aphasia therapy practice. We should therefore give an example of how this can be done. We will do so by introducing one therapy regime that has been developed over the last 20 years, variants of which have been used under different labels. The approach was developed in the context of pragmatic approaches to aphasia therapy (Aten, Caligiuri, & Holland, 1982; Bollinger, Musson, & Holland, 1993; Davis, 2005; Davis & Wilcox, 1985) and exploited ideas from the analytic philosophy of language, especially Wittgenstein’s concept of language games (Wittgenstein, 1953) and related ideas about language action relationships (see previous section headed “Semantic links”). This type of language action therapy was originally labelled “Communicative Aphasia Therapy” (Pulvermüller & Roth, 1991; Pulvermüller & Schönle, 1993). The approach was later developed further in collaboration with neuroscientists working on Constraint- Induced Motor Therapy (Miltner, Bauder, Sommer, Dettmers, & Taub, 1999; Taub, 2000; Taub et al., 1993; Taub et al., 2002). In this phase, an emphasis on massed practice and application of constraints were added. This modified approach became more well known under the label of “Constraint-Induced Aphasia Therapy” (Neininger, Pulvermüller, Elbert, Rockstroh, & Mohr, 2004; Pulvermüller et al., 2001); the expressions “forced use aphasia therapy” (Salter et al., 2005) and “constraint-induced language therapy” (Maher et al., 2006) have been used as synonyms.

A main aspect of Wittgenstein’s late philosophy was the insight that language is systematically linked to actions. To illustrate this, Wittgenstein used what he called “language games”. A famous example of such an interaction type is the so-called “Builder’s Game”, where a builder and an assistant interact in order to work on a building project. The assistant’s task is to supply the builder with building blocks of different kinds. The builder herself has to instruct the helper, thereby directing him to assist her effectively. This is best done by verbal communication, for example by using single words referring to the relevant objects or by using longer utterances. In this interaction setting, verbal actions have a clear place. Many of these speech acts are requests and must be specific enough to guarantee successful building. Wittgenstein calls these forms of communicative interactions “games”, because speech and the speech acts performed by uttering words and word strings are embedded into actions and object manipulation. This illustrates a main point of Wittgenstein’s late philosophy, that language “is woven into action”—a fact that could, as discussed above, also be verified at the level of brain circuits (for a summary of evidence, see Pulvermüller, 2005).

**Figure 3.**
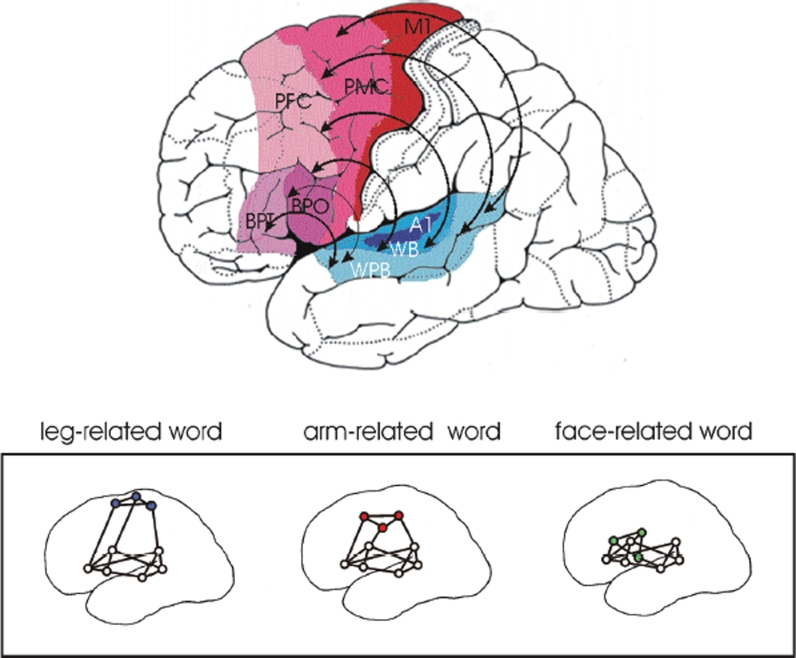
Top: Schematic illustration of long-distance links connecting the cortical areas related to language and action. Motor regions in red, inferior frontal (including Broca's) language area in purple, superior temporal (including Wernicke's) language area in blue. Bottom: A neurobiological model of language–action links: Cell assemblies processing specific language elements, for example action words, may bind information about word forms, which is laid down in perisylvian language cortex, with information about actions, which is laid down in different parts of the motor system (after Pulvermüller, 2005). Abbreviations: A1 = auditory “core” cortex, BPO = Broca, pars opercularis, BPT = Broca, pars triangularis, M1 = motor cortex, PMC = premotor cortex, PFC = posterior prefrontal cortex, WB = Wernicke, auditory belt area, WPB = Wernicke, auditory parabelt area.

The language game approach to aphasia therapy uses language games of a similar kind to implement neuroscientifically grounded principles of aphasia therapy. As language games always mimic one type of communicative interaction in everyday life, the behavioural relevance principle can be partly fulfilled. Another important aspect is that, within the language game, verbal actions, speech acts, are not used in isolation but in the context of relevant complementary actions, for example in the context of the delivery of the relevant building block or a specific kind of verbal answer (see Figure 3). The language game also makes it possible to define the type of verbal utterances that need to be produced in order to be successful. In the builder’s game itself, the types of building blocks present in the interaction determine the words and utterances that can be used to distinguish between them. Verbal demand can be constrained, for example, by introducing more, or more similar, building blocks. This illustrates one way to introduce communicative constraints. Finally, the training in a language game setting can be done frequently so that the high therapy frequency principle is also fulfilled. This shows how the three principles of maximising frequency, behavioural and communicative relevance, and focusing can be implemented.

In aphasia therapy we have explored request communications for therapeutic usage. Three patients and one therapist usually participate and interact. They sit on different sides of a square table. Each of the four participants has a set of cards in front of them and there are barriers between any two of the four to prevent them from seeing the others’ cards (Figure 3, left). Two identical sets of 16–20 cards are in the game. These are equally distributed among the players so that each of them gets 8–10 cards. For each participant the aim is to obtain as many as possible twin cards for the cards lying in front of him or her. This has to be done by communicating verbally with the other three participants.

The action structure of this language game is that of a typical request communication (Figure 3, diagram on the right). First, one of the participants makes a request addressing another participant. This can be done using one single word (e.g., “cake”) or a longer utterance of varying specificity (“the sweet thing on the plate”, “the cake please”, “could I please have the apple cake”). The sequential structure of request dialogue implies that the addressed second participant can respond in different ways to the request. He can follow the request by passing the requested card, which can be accompanied by a verbal answer (“here it is”, “please”). As a second possibility, if he doesn’t happen to have the requested card in front of him, he might want to respond by rejecting the request (“sorry, no”, “I am terribly sorry but I do not have it”). As a third alternative, he may respond by making an attempt at clarifying the request, in case there has been a comprehension or verbal communication problem (“what?”, “again please”, “can you please repeat?”). Note that apart from the words that are used for making the requests, the language skills practised in the second moves following the requests, including clarifying and rejecting, are extremely relevant in conversations between patients with language deficits and healthy individuals.

As language materials are placed in an action structure where they have their normal function as tools for making requests, rejecting them, or asking back, a high degree of behavioural and communicative relevance is being achieved. If the object pictures depict objects that are the target of request communications in everyday life, such as objects usually placed on a breakfast or dinner table, an extremely high degree of behavioural relevance, or even near-naturalness, is achieved. Constraints tailoring the communication to the needs and capabilities of the patient can be introduced through the materials. Focusing can also be provided by explicitly giving interaction rules, or by shaping and reinforcement contingency applied by the therapist. As an example for material constraints, we usually progress with severely impaired patients from pictures of objects that have names with very high lexical frequencies. Words that occur frequently in language use tend to be less impaired in patients than rare words. From there, we progress to pictures of objects whose names are less frequent and therefore usually pose a greater problem to patients with aphasia. As further steps, we introduce minimal pairs, such as “glass” and “grass”, which only differ in one phoneme, or sets of objects that are semantically and/or perceptually similar (apple cake, lemon tart, muffin), so that the set of utterances leading to communicative success is more narrowly defined. The barriers between any two of the players, which make it impossible for each of them to see the other players’ cards, should also make it less effective to use gestures, as gestures become less easily visible. By shaping and explicit rules, additional constraints can be introduced, for example the use of politeness formulas (“please”, “can you please give me”) and further material constraints can guide players to use utterances of several words. As one possibility, the cards may depict objects in different numbers and colours so that, in order to uniquely identify one specific card, it is necessary to use not only an object name but also a colour label and a number word (“please give me the three glasses with green tomatoes”). Reinforcement contingencies are applied in order to provide positive feedback for successful actions of individual partakers. These social rewards are applied in a participant-specific manner and adjusted to the performance level of each participant.

The above examples of how “constraints” are used to focus the patients on engaging in specific forms of communication should make one point even clearer. The label “constraint”, which has a negative connotation (see earlier), is used here to refer to a range of techniques that allow the therapist to guide the patient towards forms of communication he or she might otherwise avoid. This is, so to speak, a way of enriching the communicative environment of the patient and encouraging the social interactions mediated by verbal communication. Therefore the patient is not only being focused on the use of otherwise unused capabilities, reflecting the roots of this neurorehabilitation approach in the animal literature on learned non-use (Taub et al., 2002). A further point of potential importance is the enrichment of communications, in terms of additional words, utterances, speech acts, and communication sequences. This enrichment of the patient’s communications may potentially link language action therapy to established knowledge about the beneficial effects, at both neuronal and behavioural levels, of enriched environments and social interactions (Diamond, Krech, & Rosenzweig, 1964; Johansson, 1996; Johansson & Ohlsson, 1996; Risedal et al., 2002). However we must be cautious here, as any links to neuronal function are, at this point, only suggestive. A scientific proof of an effect of enriched communication triggered by language action therapy at the level of dendritic arborisation, nerve cell growth, or structural changes in cortex has as yet not been delivered.

Intensive language action therapy is applied with high therapy frequency, for example 30 hours within 10 working days. As the language game, including its actions, materials, and relevant utterances, approximates real language interaction, the principle of communicative and behavioural relevance is also fulfilled. Using the different types of constraints it also becomes possible to focus each patient on their communicative needs and possibilities in order to avoid learned non-use of verbal utterances. Language action therapy following the language game approach therefore offers a possibility to realise the neuroscientifically grounded therapy principles discussed earlier, in a pleasant and effective setting. Note that, as a therapist interacts with three patients, the therapists’ time is used very effectively

Although this section has given some details of one way of realising intensive language action therapy in the treatment of aphasia, we certainly do not suggest that this is the only way to do so. For example, other approaches to behaviourally relevant training of language (e.g., Aten et al., 1982; Bollinger et al., 1993; Davis, 2005; Davis & Wilcox, 1985) can be used as well, given there is a way to apply them successfully with high frequency and to use appropriate tools for focusing patients on their communicative needs and possibilities. It is also necessary to apply a setting that leads to similar communications repeatedly, so that the recurrence of particular language–action relationships is guaranteed. Importantly, the practising offered in the context of Constraint-Induced Aphasia Therapy was restricted to request dialogues. In other areas of pragmatic and communicative treatment, other dialogue forms have been emphasised, for example informing (Davis & Wilcox, 1985), planning, giving directions, and storytelling (Pulvermüller, 1990). Also, the use of written language has been emphasised (Meinzer, Djundja, Barthel, Elbert, & Rockstroh, 2005; Pulvermüller, 1990; Pulvermüller, Roth, & Schönle, 1992). It appears of utmost interest to widen the range of communication types in intensive language action therapy and determine efficacy and applicability to specific aphasia types for each therapeutic language game individually.

## Intensive Language Action Therapy: Efficacy

Intensive language action therapy was put to a test in a randomised controlled trial in which 17 patients were assigned randomly to either of two therapy groups. In one group language was practised in communicative and behaviourally relevant action settings. Therapy was applied with high therapy frequency and constraints instantiated by therapy materials, shaping, explicit rules, and reinforcement contingencies were applied (see earlier). In the other therapy group a more conventional structural approach to language therapy was chosen and therapy was applied for a few hours per week over a longer period of time, and therefore with lower therapy frequency. Note, however, that the same amount of therapy, the same number of therapy hours, was given to both groups (around 30 hours).

**Figure 4.**
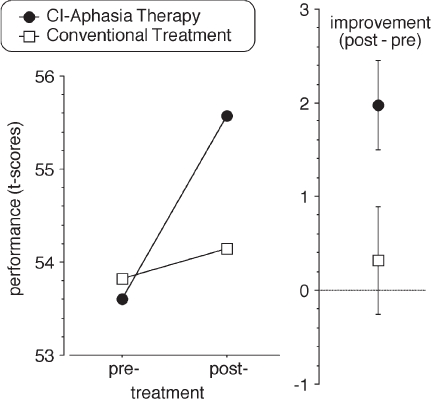
A randomised controlled trial compared one form of intensive language action therapy, Constraint-Induced Aphasia Therapy, to a less intensive conventional treatment. Although the overall number of therapy hours did not differ, the improvement (indicated separately on the right, with standard errors) achieved by patients with chronic aphasia was significantly greater for intensive language action therapy than for the control regime. T-score values calculated from clinical language tests are plotted (Pulvermüller et al., 2001).

Intensive language action therapy led to a significant increase in language performance as measured by clinical tests. The same result was achieved with a new measure, the communicative activity log or CAL, a questionnaire targeting everyday language and communication activities, which was given to the patients themselves and to independent raters (see Appendix). Some improvement, but significantly less pronounced, was found in the control group receiving the same amount of conventional therapy (Figure 4). The fact that pronounced improvements on clinical language tasks as well as on measures of everyday communication could be found as a consequence of language action therapy speaks in favour of this method.

Note also that this was one of the first studies that documented clearly significant improvements of language abilities in chronic aphasic people who had already suffered from their disease for several years on average. Previously, there had been scattered reports of therapeutically induced improvements in linguistic ability in patients with chronic aphasia after the first year of disease onset (Aten et al., 1982; Bollinger et al., 1993; Elman & Bernstein-Ellis, 1999; Katz & Wertz, 1997). In these studies, therapy was administered for many hours over an extensive period. For example, Elman and Bernstein-Ellis treated their patients for 5 hours per week for 17 weeks (85 hours; Elman & Bernstein-Ellis, 1999), and Katz and Wertz treated patients for 3 hours per week for 26 weeks (78 hours; Katz & Wertz, 1997). The totals of the numbers of therapy hours were thus more than twice the amount applied in the intensive language action therapy study (31.5 hours).

One may rightly claim that only one study of 17 patients demonstrating an effect of the new therapy approach is probably too little evidence to allow for generalised conclusions (see Salter et al., 2005). Therefore, it is important to point to replications that have been undertaken since the first publication. Meinzer and colleagues performed such a study demonstrating that a larger number of patients (24) benefited from intensive language action therapy (Meinzer et al., 2005). In this study, additional minor benefits were suggested that potentially relate to massive practising by aphasia patients and their relatives, which was done in addition to language therapy led by trained therapists. Based on previous work, it was expected that practising led by volunteers would achieve improvements comparable with those induced by professional treatment (Marshall et al., 1989; Wertz et al., 1986). The important contribution of this work is that the authors not only could replicate the effect of intensive language action therapy, but could also demonstrate for the first time that this approach leads to stable effects persisting for ~,6 months after therapy.

Maher and colleagues compared Constraint-Induced Aphasia Therapy to a treatment approach allowing all communication modalities. Importantly, equally high-frequent training was given with both therapy methods (Maher et al., 2006). The authors suggest that participants showed more consistent improvement on standard aphasia measures and clinician judgements of narrative discourse after Constraint-Induced Aphasia Therapy with focus on communicative needs, compared with the approach offering free choice of communication modalities. The study by Maher and colleagues includes the first evidence that, apart from quantity and frequency of practice and behavioural relevance, the third therapy principle, focusing, has a beneficial effect of its own.

These and similar results provide strong evidence that language action therapy— carried out in a massed practice fashion, emphasising language use in action context, and applying communicative constraints to focus the patients on their communicative needs and capabilities—is an effective tool in improving language abilities in chronic aphasia patients. The classical wisdom had once been that language recovery approaches ceiling after half a year or one full year after onset of the disease. Research on neuroscientifically grounded therapy in the language–action context now demonstrates that this is not the case. If there is a ceiling after 1 year post disease onset, this ceiling can be removed by applying therapy guided by neuroscience knowledge.

Still, we do not know which contribution each of the neuroscientific principles discussed makes to therapy success. Is it most relevant to provide therapy in a high- frequency fashion, so that high therapy frequency, a maximum of the number of treatment hours per time interval, is achieved? Or is it more important that behavioural relevance, the practising of language used in action contexts and everyday language settings, is guaranteed? Finally, how important is it to provide strong focusing through constraints by means of materials, shaping, explicit introduction of rules, and reinforcement contingencies? So far, evidence is strongest that the triple pack of principles leads to successful outcome. Future studies may explore further the contribution of each principle specifically. However, as each of the three principles is well grounded in established neuroscience knowledge, it appears advisable to apply all three principles together. Should strong evidence emerge for the irrelevance of one of the principles, a modification might be considered. It should also be noted that not only could progress be documented using clinical language tests, therefore demonstrating an improvement of the deficits that define aphasic syndrome: in addition, the assessment of functional communication using, for example the Communicative Activity Log (Pulvermüller et al., 2001), carried out by patients, their relatives, and by independent raters blind to the hypotheses of the study and the group assignments, all showed that the communication impairment resulting from the aphasic syndrome improved too. For convenience, the Communicative Activity Log or CAL is given in full length in the Appendix. In future, it will be important to explore also the social, psychosocial, and emotional consequences of intensive language action therapy and their impact on the patients’ day-to-day life. This is possible using procedures that appropriately assess these variables (e.g., Code & Müller, 1992; Code, Müller, Hogan, & Herrmann, 1999; Hemsley & Code, 1996).

## Drugs and Trends

We have reviewed new approaches to aphasia therapy taking advantage of neuroscience knowledge. Principles derived from neuroscience research emphasise massing of practice, behavioural relevance of the training, and focusing of communicative actions on the patients’ needs and possibilities. Neuroscientifically grounded aphasia therapy is still in its infancy, and there are, of course, multiple ways of improving the currently successful approaches in this domain. Therefore it is, as we believe, important to think about optimising these current methods. The present section will focus on this issue.

### Perspectives of intensive language action therapy

Here are a few obvious suggestions to optimise language action therapy along dimensions defined by the major neuroscience principles of massing, behavioural relevance, and focusing.

If the amount of therapy hours applied is kept constant, a greater therapy frequency improves the outcome (Bhogal et al., 2003; Pulvermüller et al., 2001). This suggests that condensing therapy to an even greater frequency of above 3 hours per day may lead to additional benefits. As therapy by professional speech therapists is usually limited in time and intensity (Katz et al., 2000), due to a range of practical and financial reasons, additional training of patients by non-professionals (Basso, 2005; Basso & Caporali, 2001; Marshall et al., 1989; Wertz et al., 1986) can offer a route to increasing therapy frequency. Structured training of relatives, caregivers, and other regular interaction partners of patients with aphasia might therefore become a valuable target of future research. A further strategy to increase the number of practice hours, along, possibly, with therapy frequency, is offered by the use of computers in aphasia therapy. Computer programs mimicking aspects of therapeutic language games are already available (e.g., Pulvermüller et al., 1992; Roth & Schönle, 1992), and it is well known that computer training can have a beneficial effect on aphasia recovery (Stachowiak, 1987; Wertz & Katz, 2004). It may therefore be feasible to complement language action therapy with computer intervention, so further increasing amount of therapy and frequency.

Given the recent advance in the development of speaking and interacting robots (Roy, Hsiao, & Mavridis, 2004; Wermter & Elshaw, 2003; Wermter et al., 2004), it even appears feasible to suggest the development of communication robots for intensive language action therapy in future. The agent’s action repertoire, visual perceptual classification skills, and language analysis and production systems would be tailored to the requirements of specific language games (see previous section for an example). The agent’s role would also include that of a communication assistant offering help and suggestions in case the patient is unable to communicate in a given context. Availability of such agents could open new dimensions for intensive language action therapy, as the present limits of massing practice could be extended substantially.

What has been said about the possible optimisation along the therapy frequency axis can also be generalised to the other principles, behavioural relevance and focusing. Behavioural relevance and communicative significance can be achieved by a range of treatment approaches in the pragmatic domain (Aten et al., 1982; Bollinger et al., 1993; Davis, 2005; Davis & Wilcox, 1985). Here, a preference might be given to those approaches that allow for repeated practising of utterances and speech acts. Note that, for example in role-playing approaches, it is difficult to implement such repetition and therefore effective correlation between relevant learning events is not easy to establish. The degree to which the communications in therapy are tailored to the individual person’s needs is also a variable worth exploring. It can be varied, for example, by adding single patient treatment regimes to the group therapy approach discussed here. Single patient treatment may allow a more specific adjustment of language games to the individual’s needs than is possible in a group setting (see, for example, Pulvermüller & Schönle, 1993). If, after a substantial period of training, a ceiling has been reached for certain patients, it may also be fruitful to introduce alternative and augmented communication strategies into the therapeutic settings (Waller, Dennis, Brodie, & Cairns, 1998).

### Perspectives opened by drugs

Drugs can be used to facilitate recovery from stroke and other diseases of the central nervous system. The notion of using drugs to improve language deficits is relatively new and still controversial, mainly because the rationale for early pharmacological interventions had not been justified at a theoretical level. However, current motivations for using drugs in aphasia therapy build on strong evidence from neuroscience that agents acting on specific neurotransmitter systems can modulate neuronal function of these systems in well-defined ways, ranging from mimetic or competitive and non-competitive inhibitory effects to facilitation of neuronal plasticity and long-term potentiation. If a lesion impairs the functionality of neuronal circuits contributing to language, such modulation of neuronal functionality can have a beneficial effect on language performance (Albert, Bachman, Morgan, & Helm-Estabrooks, 1988; Berthier, 2005; Shisler, Baylis, & Frank, 2000).

In the past few years, a range of pharmacological intervention studies of aphasia have been undertaken. Pharmacotherapy was either given as the only therapy or in conjunction with speech-language therapy. In the latter type of study, where agents acting on transmitter systems affected by focal brain injury are given on top of behavioural treatment, a beneficial effect of pharmacotherapy on performance was documented on a range of language tasks (including spontaneous speech, naming, repetition, auditory comprehension) (Berthier, 2005; Parton, Coulthard, & Husain, 2005; Shisler et al., 2000; Small, 2004). A variety of pharmacological agents has already been explored in the therapy of aphasia and results indicate that the application of some of these agents has clearest beneficial effect either specifically on language recovery or, more generally and indirectly, by improving arousal, attention and working memory necessary for successful language processing (Table 1).

**TABLE 1:** Summary of effects of pharmacotherapy in the treatment of post-stroke aphasia

*Agent*	*Neurotransmitter*	*Study design*	*Number of studies*	*Main results*
Bromocriptine	Dopamine	Single cases Case series Open-label RCT	11	Positive effects in open label studies mainly in transcortical motor aphasia and a dynamic aphasia of moderate severity. Improvement in overall aphasia severity in chronic patients. Variable outcomes in Broca's aphasia. Lack of efficacy in severe cases. Various RCTs negative.
Piracetam	GABA, Excitatory aminoacids	RCT	7	Positive effects in the acute stage in overall language measures, spontaneous speech and written language. Lack of efficacy in chronic stages.
Donepezil	Acetylcholine	Single cases Case series Open label RCT	6	Positive effects in global aphasia severity, communication, spontaneous speech, comprehension, naming and speed and accuracy of information processing. Efficacy maintained at long-term follow-up.
Dexanfetamine	Norepinephrine Dopamine Serotonine	Open-label RCT Single-case RCT	3	Positive effect in overall performance on communication in subacute stages with efficacy maintained at chronic stages. Negative effect in anomia in a single-subject RCT.
Bifemelane	Acetylcholine	RCT	1	Positive effect on anomia.
Fluvoxamine	Serotonine	Crossover	1	Positive effect on anomia, perseverations and mood in fluent aphasia.
Moclobemide	Serotonine Norepinephrine Dopamine	RCT	1	Negative effect.
Zolpidem	GABA	Single case	1	Positive effect, transient effect on verbal output

*Notes:* Only agents with a theoretically-driven rationale to be used in the treatment of aphasia were included. Other agents (e.g., haloperidol, propranolol, thiazide diuretics, chlordiazepoxide) lacking scientific justification were excluded. GABA indicates γ aminobutyric acid; RCT = randomised controlled trial. See further details and references of pharmacological studies in Berthier, 2005; Shisler et al., 2000; Small, 2004.

It may be that specific approaches to aphasia therapy at the behavioural level interact with changes at the neurochemical level. In this case, the best outcome of a behavioural treatment approach may be achieved if neuronal activity is modified by neurochemical agents during the behavioural treatment period. In this context, a number of drugs are potentially relevant and all of them have their major effect in maintaining a dynamic signalling process between neurons. Excitatory synapses of the cortex mainly use glutamate as their transmitter. In pathological conditions, such as focal brain injury, there is a failure to regulate the concentration of glutamate with the potential risk of causing cell toxicity and death. A major problem in cortical functioning after, for example, a stroke, arises from over-activation in the periphery of the lesion (e.g., ischaemic penumbra). It may be beneficial, in this case, to reduce the overall level of cortical activation in these areas by using agents that inhibit glutamate at its receptors, thereby restoring the physiological equilibrium of neurotransmission at the glutamate synapse. In essence, it may therefore be beneficial to inhibit glutamate at its synapses to achieve improved functioning (Berthier, 2005; Parton et al., 2005). Our own preliminary results indicate that memantine, a non-competitive *N-methyl*-D-*aspartate receptor* antagonist, may benefit language and verbal memory functions in patients with chronic aphasia, especially if intensive constraint-induced aphasia therapy is provided at the same time (see Table 1). Therefore, memantine and other glutamate inhibitors in connection with intensive language action therapy might open perspectives towards improving aphasia therapy further.

Apart from glutamate, other neurotransmitters and their synapses have been in the focus of pharmacological treatment of language disturbances. The relevant neurotransmitters include the monoamines dopamine, norepinephrine, and seroto- nine, and also acetylcholine. These neurotransmitters are mainly produced in cells in the mid-brain and basal forebrain and exert their function in basal ganglia, thalamus, and neocortex. Dopamine agonists, for example bromocriptine, do not appear to have a beneficial effect on the amelioration of aphasia (Berthier, 2005; Bragoni et al., 2000; Gupta, Mlcoch, Scolaro, & Moritz, 1995; Sabe, Salvarezza, Garcia Cuerva, Leiguarda, & Starkstein, 1995), unless nonfluent output is a consequence of decreased drive to generate speech. This appears to be the case in adynamic aphasia and transcortical motor aphasia, where intervention with bromocriptine is beneficial (Berthier, 1999, 2005; Raymer, 2003; Raymer et al., 2001). However, various randomised controlled trials (RCT) of bromocriptine in aphasia were negative (see above; Berthier, 2005).

The efficacy and safety profile of piracetam, a compound acting on GABA-ergic and cholinergic systems as well as on excitatory aminoacids, have been examined in various randomised controlled trials. Although results consistently indicated that piracetam is effective when administered in the acute and subacute phases of stroke, especially if applied during behavioural language treatment (Enderby, Broeckx, Hospers, Schildermans, & Deberdt, 1994; Huber, Willmes, Poeck, Van Vleymen, & Deberdt, 1997; Kessler, Thiel, Karbe, & Heiss, 2000; Szelies, Mielke, Kessler, & Heiss, 2001), its efficacy vanished in the chronic stages, thus precluding the maintenance of treatment in patients with chronic aphasia.

Mixed evidence exists for the effect of monoaminergic drugs. However, so-called MAO (mono-amino-oxidase) inhibitors, which are used as antidepressants in the clinic and temporarily enhance serotonine, norepinephrine, and dopamine levels at their respective synapses, could not be shown to improve language performance in aphasia patients (Laska, von Arbin, Kahan, Hellblom, & Murray, 2005). Still, serotonine and nonepinephrine reuptake inhibitors (especially amphetamine and its derivatives), which are known to temporarily enhance attention and arousal levels, can have a beneficial effect of language abilities in aphasia patients given that they are applied during behavioural language treatment (Walker-Batson et al., 2001). The selective serotonine reuptake inhibitor fluvoxamine with antidepressant properties significantly improved naming, perseverations, and mood in stroke patients with fluent aphasia (Tanaka et al., 2001). Interestingly, monoaminergic drugs, including amphetamines and also Levodopa, which metabolises into the transmitter dopamine after crossing the blood–brain barrier, have been shown to facilitate word learning in normal healthy subjects (Breitenstein et al., 2006; Breitenstein et al., 2004; Knecht et al., 2004). This further suggests that monoaminergic treatment might also have beneficial effects on language learning in patients.

Donepezil, a cholinesterase inhibitor with a selective central action, appears to be more promising than other drugs to treat aphasia even in chronic stages (Berthier, Hinojosa, Martin, & Fernandez, 2003). Donepezil, which enhances acetylcholine levels at the synapses by inhibiting the enzyme (acetylcholinesterase) that normally degrades it, even had an effect in chronic patients (Berthier, Hinojosa, Martin Mdel, & Fernandez, 2003). Moreover, a recent controlled trial combining donepezil with standard speech-language therapy in chronic post-stroke aphasia replicated not only the results of our own open label trial on measures of overall aphasia severity, but also further demonstrated significant improvements in communication skills and processing speed and accuracy (Berthier & Green, 2007; Berthier et al., 2006a). That this latter drug, donepezil, might interact with neuroscientifically grounded behavioural therapy is suggested by a study of its combined effect with Constraint-Induced Motor Therapy (Nadeau et al., 2004). There is limited evidence for effects of other cholinergic treatments on aphasia (Tanaka, Miyazaki, & Albert, 1997).

Taken together, this evidence indicates that a range of drugs acting on neurotransmitter metabolisms can influence cortical functioning in a way that helps patients with aphasia improve their language skills (Berthier, 2005; Parton et al., 2005). At this point, glutamatergic, monoaminergic, and cholinergic drugs seem to be most promising (see Table 1). It appears to be a fruitful area of future research to define the strengths and weaknesses of each of the possible drug treatments. Of utmost importance is research into the interactive effects of these drugs when applied together with behavioural language treatment of different types, and, especially, together with neuroscientifically grounded intensive language action therapy.

## From Therapy Back to Neuroscience: Investigating Functional Reorganisation of Language Circuits

In previous sections, we argued that neuroscience has had, and will have, a major influence on aphasia therapy. Here, in the last part of our paper, we will ask whether the route between neuroscience and therapy is one-way. The answer will be negative: Neuroscience research can receive important input from the study of aphasia therapy, especially (i) if therapy is applied to patients in a chronic stage of the disease and (ii) if it can be shown to be effective in a short time.

Functional reorganisation after lesions of the brain has become a major topic of neuroscience research. Motor skills and even language and other higher cognitive functions that fall victim to a stroke or other disease of the central nervous system can, after some time, show restitution, so that the patients regain to different degrees their ability to use the function. During the period of restitution, brain activity may show characteristic changes, which can then be tentatively attributed to the brain processes supporting restitution of functions. Unfortunately, however, the interpretation of spontaneous restitution in terms of reorganisation processes is complicated by the fact that restitution is overlaid by organic healing processes, for example the reduction of oedema and the reperfusion of critical cortical areas along with social and emotional changes. It is well documented that the degree of perfusion of brain areas not directly damaged can be reflected in behavioural language deficits (Hillis et al., 2004; Hillis et al., 2006). Therefore it is impossible to determine with certainty whether any behavioural changes observed in acute patients are a by-product of the well-known spontaneous restitution processes of the nervous tissue or, critically, are a manifestation of a functional behavioural change and represent an underlying modification of neuronal circuits; that is, neuronal reorganisation. In this context, it is important to distinguish spontaneous restitution processes (what Monakow called “diaschisis”, von Monakow, 1914) from neuronal reorganisation. Neuroscience studies of the plasticity of the brain processes of cognition and language would naturally target the latter; that is, the neuronal reorganisation processes, or modification of nerve cell circuits underlying specific brain functions.

In stroke patients, the period during which spontaneous restitution usually takes place is the first 6–12 months after onset of the disease (Rosenbek et al., 1995). After this period, behavioural parameters and clinical test results usually remain stable. To differentiate between spontaneous restitution and neuronal reorganisation processes, it is necessary to perform studies of cortical reorganisation with chronic patients for whom this critical period has already passed (see also earlier). Unfortunately, however, behavioural changes are very difficult to achieve after the period of spontaneous recovery. If such changes at a chronic stage occur at all, they require lengthy treatment. This is a major problem, as during a long interval of several months to years, behavioural, emotional, and social changes unrelated to therapy may take place in chronic patients. In prolonged therapy of chronic patients there is therefore a danger of falsely attributing such therapy-unrelated changes to cortical reorganisation. To draw safe conclusions on therapy-related changes in cognitive and language processing and reorganisation, it is therefore imperative to investigate behavioural and neuronal correlates of rapid improvement brought about by short intervals of therapy in chronic patients.

It is here that the newly developed methods for intense language action therapy become significant in neuroscience research. As reviewed in previous sections, these methods can lead to improvements of language skills within a short period of a couple of weeks. As we have also emphasised earlier, these methods lead to significant language improvements in chronic patients for whom normally no spontaneous recovery processes can be expected. Intensive language action therapy in patients with chronic aphasia may therefore be an ideal tool for investigating cortical reorganisation processes of language circuits in the human brain.

**Figure 5.**
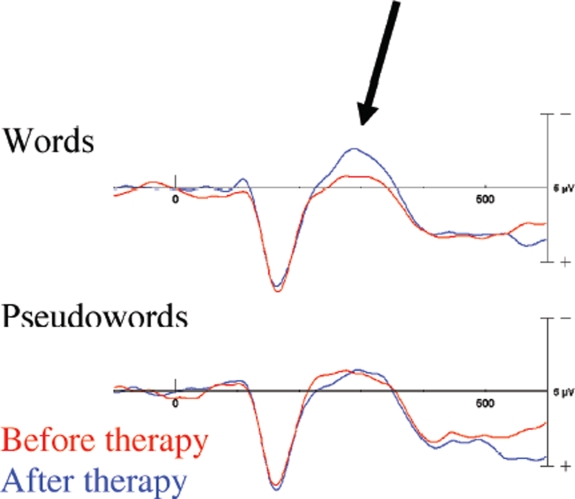
Neurophysiological changes induced by intensive language action therapy in chronic aphasic participants. A brain potential elicited by written words at a latency of ∼250 ms increased significantly over a short therapy period. No comparable change was seen for meaningless pseudowords. The word-specific increase of this “Aphasia Recovery Potential” correlated with the improvement on a clinical language test and its sources were localised in both cortical hemispheres (after Pulvermüller et al., 2005b).

In a study of stroke patients with chronic aphasia, intensive language action therapy was applied for 2 weeks, yielding a significant improvement of language performance as assessed by clinical tests (Pulvermüller et al., 2005b). Neurophysiological activity elicited by words and pseudowords was measured before and after treatment. After the therapy interval, word-evoked potentials (a negativity with latency 250–300ms) became significantly stronger, whereas responses to meaningless pseudowords did not change. Word-specific changes were documented by analysis of event-related potential amplitudes and root mean square values, which revealed interactions of the factors assessment time (before versus after therapy) and lexicality (word versus pseudoword). Source localisation using minimum norm current estimation showed that bilateral cortical sources activated by word stimuli contributed to the change, demonstrating that neuronal networks distributed over both hemispheres were the substrate of cortical reorganisation of language processes in intensive aphasia therapy. Word-evoked differences source strengths were significantly correlated with performance on a clinical language test (Token Test; De Renzi & Vignolo, 1962), demonstrating a link between behavioural and neurophysiological changes (see Figure 5). We suggest that the early word evoked negativity might represent an index of reorganisation of language after stroke; that is, an aphasia recovery potential.

Earlier work on brain dynamics following aphasia used PET and fMRI measuresto assess functional changes in patients, or just activation differences between patients with aphasia and normal controls. As mentioned earlier, a number of these studies suggested a main contribution of the left hemisphere to language-related reorganisation (Heiss et al., 1999; Price & Crinion, 2005), whereas another set of studies argued in favour of a major role of the right hemisphere in language reorganisation (Abo et al., 2004; Blank et al., 2003; Musso et al., 1999; Thomas et al., 1997; Weiller et al., 1995). Some of this work documented a difference in laterality of brain activation in aphasia patients compared with normal controls during cognitive and language tasks. Such comparisons are limited, as the changes in behaviour together with those in brain activation (e.g., its laterality) are not a direct result of a lesion, but are under the influence of the strategies applied in the cognitive and language tasks (Dobel et al., 2001). For most earlier studies looking at changes over time or over therapy, patients were examined and/or treated shortly after their stroke, within the first year, during which it is well known that spontaneous restitution is still pronounced and may substantially vary between patients. These studies therefore suffer from the mutual confounding of spontaneous restitution, due to reperfusion and shrinking of oedema, etc., and, on the other hand, neuronal reorganisation; that is, the reformation of language circuits. Only in a minority of studies have chronic patients been monitored, but in this case it was necessary to perform lengthy therapy over several months, so that changes along social, emotional, communicative strategic, or clinical psychological dimensions could have contributed to the behavioural and neuronal changes observed. As intensive language action therapy makes it possible to improve language performance and abilities within a short period of time in chronic patients, application of this method may provide a superior strategy for monitoring the build-up of language circuits after brain lesion.

Even though the conclusions suggested by the study on the neurophysiological reorganisation during intensive language action therapy are still limited, results clearly support a role of sources in both cortical hemispheres in the improvement of word processing (Pulvermüller et al., 2005b). We note again that, from the clinical literature, it is well known that not only the left hemisphere but also the right can support language, and that there is even compelling experimental evidence for involvement of both hemispheres and for facilitatory hemispheric interaction in normal language processing (see above). Therefore, we believe that a bi-hemispheric contribution to language reorganisation is in good agreement with the literature. Further evidence comes from work directly supporting dynamics in both hemispheres that relate to aphasia or aphasia therapy (Cardebat et al., 2003; Crosson et al., 2005; Dobel et al., 2001).

Views on specific contributions of the left dominant and right non-dominant hemispheres to language reorganisation can be integrated within a bi-hemispheric interactive perspective, building on the concept of transcortical cell assemblies that include neurons in both hemispheres (Pulvermüller & Mohr, 1996). In this sense, language-related neural systems distributed over both hemispheres may therefore improve their internal connections, thus leading to stronger and faster activation processes after therapy than before. Both hemispheres would thus take their shares in providing the substrate for the strengthening of neuronal connections that underlies language reorganisation in chronic aphasia.

## Summary

We here summarised progress in theoretical neuroscience and research on language processing in the human brain and highlighted implications of this knowledge for the practical domain of aphasia therapy. We reviewed neuroscience evidence on learning at the neuronal level, on brain links of the cortical systems for language, action, and perception, and on post-injury neurobehavioural processes of the chronification of neurological dysfunction. From this neuroscience research we deduced three principles of aphasia therapy, recommending (i) *massing of training*, (ii) *behavioural and communicative relevance* of the interaction during treatment, and (iii) *focusing* of training on the patients’ communicative needs and possibilities. These principles are condensed into a new family of treatment approaches, which we here tag “intensive language-action therapy”, or ILAT. An exemplification followed, showing how the three principles can be realised in the context of Constraint-Induced Aphasia Therapy, to yield one form of intensive language action therapy. Work proving the efficacy of this new approach to aphasia therapy was reviewed and perspectives for improving ILAT, and neurocognitive therapy in general, were highlighted. Special attention was paid to perspectives offered by conjunctive treatment applying language action therapy together with neuropharmacological treatment. Finally, we claimed that therapy studies using ILAT to treat chronic aphasia patients have a unique place in theoretical cognitive neuroscience research. They are indispensable for clarifying the plastic brain processes of the reorganisation of language in the human brain, especially for separating them from restitution processes. Preliminary evidence for a bi-hemispheric involvement for such “pure” language reorganisation in patients with chronic aphasia was discussed in closing.

## Appendix

### Communicative Activity Log (CAL) after Pulvermüller, Neininger, Elbert, Mohr, Rockstroh, Koebbel, & Taub (2001)

The CAL can be administered to obtain information about a patient’s communicative behaviour in everyday life. A range of speech acts and features of communicative interactions are monitored. Each version of the CAL includes items addressing speech output and language comprehension. The two parts of the CAL address quality and quantity of communication. The CAL version that appears below is for evaluation of a patient’s communication performance by a therapist, clinician, relative, or independent observer. A self-evaluation questionnaire, which can be read to patients with mild aphasia, is derived from this questionnaire by replacing the words “the patient” by “you” in each question (after Pulvermüller et al., 2001).

To obtain the CAL score, the ratings are converted into points, according the scheme given below. The CAL *quality* score is obtained by summing up scores for items 1–18. The *quantity score* is obtained by summing up scores over items 19–36.

#### Quality of communication

1. How well would the patient communicate with a relative or good friend?
  Never – with major problems – with minor problems – well at basic level – well at moderate level – extraordinarily well
2. How well would the patient communicate when together with a group of friends or relatives?
  Never – with major problems – with minor problems – well at basic level – well at moderate level – extraordinarily well
3. How well would the patient communicate with a foreigner?
  Never – with major problems – with minor problems – well at basic level – well at moderate level – extraordinarily well
4. How well would the patient communicate when in a group together with several others he or she does not know?
  Never – with major problems – with minor problems – well at basic level – well at moderate level – extraordinarily well
5. How well would the patient communicate in an office, store or public institution (post office, butcher etc.)?
  Never – with major problems – with minor problems – well at basic level – well at moderate level – extraordinarily well
6. How well would the patient communicate on the telephone?
  Never – with major problems – with minor problems – well at basic level – well at moderate level – extraordinarily well
7. How well would the patient understand to news on the radio or TV?
  Never – with major problems – with minor problems – well at basic level – well at moderate level – extraordinarily well
8. How well would the patient understand the content of a newspaper article?
  Never – with major problems – with minor problems – well at basic level – well at moderate level – extraordinarily well
9. How successful would the patient be when writing down short notes?
  Never – with major problems – with minor problems – well at basic level – well at moderate level – extraordinarily well
  **Table:**
  *Judgement of quality*	*Judgement of quantity*	*Number of points*
  Never	Never	0
  With major problems	Almost never	1
  With minor problems	Rarely	2
  Well at basic level	Sometimes	3
  Well at moderate level	Frequently	4
  Extraordinarily well	Very frequently	5
10. How well would the patient solve simple arithmetic problems?
  Never – with major problems – with minor problems – well at basic level – well at moderate level – extraordinarily well
11. How well would the patient communicate when under stress?
  Never – with major problems – with minor problems – well at basic level – well at moderate level – extraordinarily well
12. How well would the patient communicate when relaxed and not under stress?
  Never – with major problems – with minor problems – well at basic level – well at moderate level – extraordinarily well
13. How well would the patient communicate when he or she is tired?
  Never – with major problems – with minor problems – well at basic level – well at moderate level – extraordinarily well
14. How well would the patient make statements or reports about facts?
  Never – with major problems – with minor problems – well at basic level – well at moderate level – extraordinarily well
15. How successful would the patient be at asking a question?
  Never – with major problems – with minor problems – well at basic level – well at moderate level – extraordinarily well
16. How well would the patient answer questions asked by others?
  Never – with major problems – with minor problems – well at basic level – well at moderate level – extraordinarily well
17. How well would the patient verbally express criticisms or make complaints?
  Never – with major problems – with minor problems – well at basic level – well at moderate level – extraordinarily well
18. How well would the patient verbally respond to criticisms?
  Never – with major problems – with minor problems – well at basic level – well at moderate level – extraordinarily well

#### Amount of communication

19. How frequently would the patient communicate with a relative or good friend?
  Never – almost never – rarely – sometimes – frequently – very frequently
20. How frequently would the patient communicate when together with a group of friends or relatives?
  Never – almost never – rarely – sometimes – frequently – very frequently
21. How frequently would the patient communicate with a foreigner?
  Never – almost never – rarely – sometimes – frequently – very frequently
22. How frequently would the patient communicate when in a group together with several others he or she does not know?
  Never – almost never – rarely – sometimes – frequently – very frequently
23. How frequently would the patient communicate in an office, store or public institution (post office, butcher etc.)?
  Never – almost never – rarely – sometimes – frequently – very frequently
24. How frequently would the patient use the telephone?
  Never – almost never – rarely – sometimes – frequently – very frequently
25. How frequently would the patient listen to news on the radio or TV?
  Never – almost never – rarely – sometimes – frequently – very frequently
26. How frequently would the patient read the newspaper?
  Never – almost never – rarely – sometimes – frequently – very frequently
27. How frequently would the patient write down short notes?
  Never – almost never – rarely – sometimes – frequently – very frequently
28. How frequently would the patient solve simple arithmetic problems?
  Never – almost never – rarely – sometimes – frequently – very frequently
29. How frequently would the patient communicate when under stress?
  Never – almost never – rarely – sometimes – frequently – very frequently
30. How frequently would the patient communicate when relaxed and not under stress?
  Never – almost never – rarely – sometimes – frequently – very frequently
31. How frequently would the patient communicate when he or she is tired?
  Never – almost never – rarely – sometimes – frequently – very frequently
32. How frequently would the patient make statements or reports about facts?
  Never – almost never – rarely – sometimes – frequently – very frequently
33. How frequently would the patient ask a question?
  Never – almost never – rarely – sometimes – frequently – very frequently
34. How frequently would the patient answer questions asked by others?
  Never – almost never – rarely – sometimes – frequently – very frequently
35. How frequently would the patient verbally express criticisms or make complaints?
  Never – almost never – rarely – sometimes – frequently – very frequently
36. How frequently would the patient verbally respond to criticisms?
  Never – almost never – rarely – sometimes – frequently – very frequently
